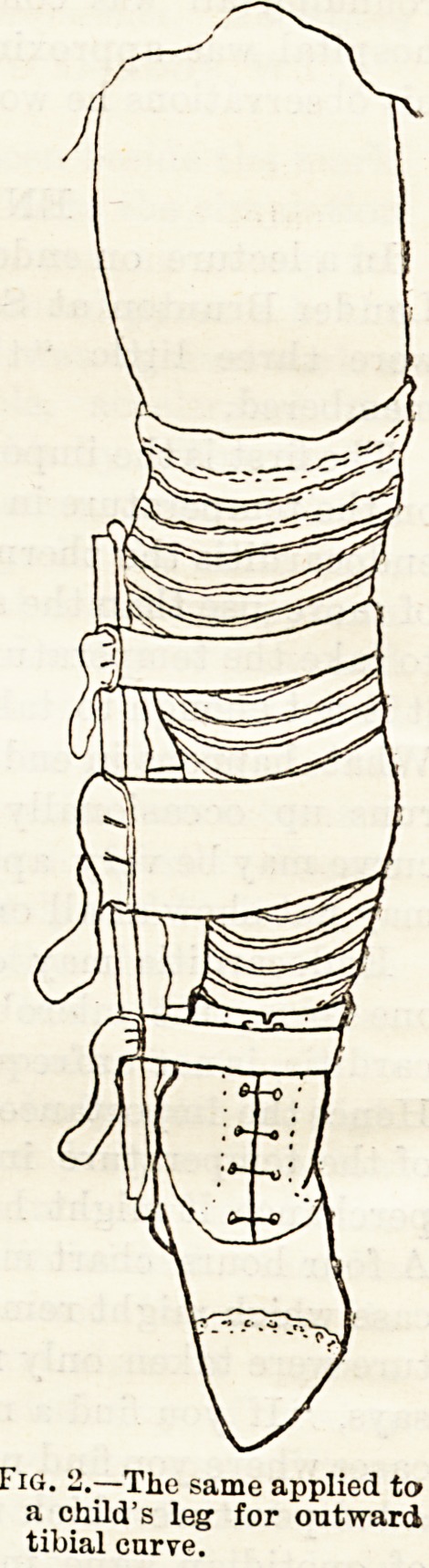# Practical Observations on the Treatment of Common Deformities Caused by Rickets
*An address given at the City Orthopædic Hospital, May 6th, 1897.


**Published:** 1897-06-19

**Authors:** J. Jackson Clarke

**Affiliations:** Assistant-Surgeon at the North-West London and City Orthopædic Hospitals


### PRACTICAL OBSERVATIONS ON THE TREATMENT OF COMMON DEFORMITIES CAUSED BY RICKETS.*

By J. Jackson Clarke, M.B.Lond., F.R.C.S., Assistant-Surgeon at the North-West London and City Orthopædic Hospitals.

The importance of the early treatment of rachitic deformities is only fully realised in institutions where great numbers of deformed patients are gathered together. Among cases of disability to earn a living from severe deformity, whether of spine, chest, or limbs, those due to rickets constitute a considerable section. To the individual, to the family, and to the State, rickets is a heavy burden. The incidence of the disease is chiefly upon the poor, but owing largely to the advertising energy of the proprietors of artificial foods, it is by no means uncommon among the well-to-do. It has been to me a happy experience that if the deformities are treated in their earlier stages they may be cured without difficulty and without curtailing the freedom of movement so essential to the healthful development of children. I need not dwell long upon the anatomical changes observed in the bones. They are best realised by comparing a vertical section of a normal long bone during the period of growth with a similar section of a long bone showing typical enlargement at the epiphyseal ends of the shaft or diaphysis. In the normal bone the meeting of diaphysis and epiphysis is marked by an even zone which measures less than one-tenth of an inch in depth, and consists of an opaque white portion on the side of the shaft; this is caused by the calcification of the remaining bars of the matrix of the epiphyseal cartilage and the formation of bone on their outer sides. An upper part of the ossifying zone is translucent and represents the extent of the proliferation of cartilage cells towards the diaphysis. Comparing this with what is seen in a long bone with well-marked rachitic change, it is seen that the even double zone of less than one-tenth of an inch in depth is replaced by an area of half an inch or more in depth and bulging at the sides. In the epiphyseal third this area is translucent, and consists of proliferating cartilage which towards the diaphysis is prolonged into irregular processes and insulated fragments. The middle part of the area is composed of cartilage intersected by irregular processes of vascular marrow prolonged from the shaft and surrounded by osteoid tissue. A third part contains slender spicules of bone, and is an exaggerated degree of the normal ossification about the remains of the epiphyseal cartilage. Thus what should be a narrow regular zone of less than one-tenth of an inch in a marked case of rickets is more than twenty times this depth, and by bulging at the sides causes the thickening which constitutes the "double-jointed" condition of the bones of the limbs and the "beading" of the ribs. Sometimes there is an excessive formation of soft bone from the periosteum, and in all cases of severe rickets all parts of the bones are softer than normal because the ratio of soft vascular marrow to hard bony tissue is altered in favour of the soft marrow, and much of the tissue that should be hard bone is limeless bone or osteoid tissue. I can now define what I mean by the

* An address given at the City Orthopædic Hospital, May 6th, 1897.

early stages of rachitic deformity, the treatment of which will be illustrated by cases now under my care. In the case of the long bones I do not, of course, refer to the swellings near their articular ends, but to angular deformities in the shafts, aided in cases, such as knock-knee, by alterations in the ligaments. Before a child begins to walk rachitic deformities in the spine and thorax most frequently call for treatment, whilst after walking has commenced deformities of the lower extremity are commonly more in need of treatment. Examples of rachitic deformity of the lower extremity may be taken.

**Outward Tibial Curve.**—Case 1. A little boy, aged four years, suffering from one of the most frequent forms of bow-leg; that is, from an outward bending of the shaft of the tibia. He is wearing the simple inside splints, which reach upwards just above the knee, and downwards to the sole of the foot. They are slightly hollowed, and are fixed by three bands of webbing—one below the knee, one at the ankle, and another opposite the middle of the curve. The upper and lower bands are 1¼ in. in width, the middle one is broader, 2¼ in. An illustration will best show the character of the splint, and since the success of the treatment depends upon accurate attention to detail, even this simple splint, which was devised by the late E. J. Chance, and has been used for many years by my senior colleagues—Noble Smith and John Poland—at the City Orthopædic Hospital, demands close study.

Figs. 1 and 2 show the chief features of the splint. The padding is stitched only across the middle portion of the splint, so that the smaller bands can be passed between the padding and the splint, and made to encircle the latter at the notches, to prevent their becoming displaced. After the apparatus has been adjusted to the limb, the broad central band may be stitched to the webbing so that it does not slip away from the convexity of the bend. This middle band is the one that effects the straightening of the bone, and in the course of the day it requires tightening from time to time. At bedtime the splint should be taken off, and, after the limb has been washed and rubbed, it should be reapplied and kept on all night. The great advantage of this simple apparatus is that it allows the little patient to walk and run during the term of treatment.

Fig. 1.—Chance's splint for outward tibial curve.

Fig. 2.—The same applied to a child's leg for outward tibial curve.

*(To be continued.)*

## Figures and Tables

**Fig. 1. f1:**
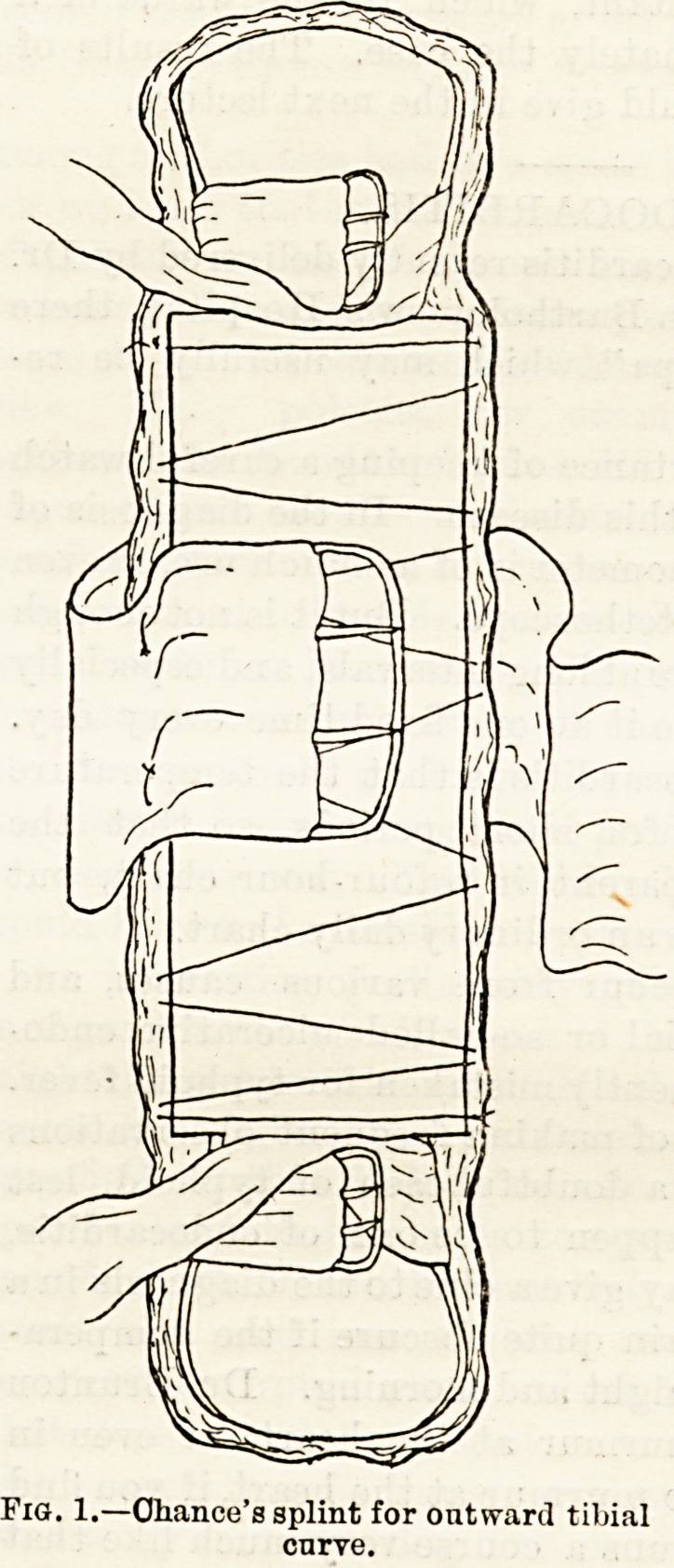


**Fig. 2. f2:**